# Effects of a gratitude intervention program on work engagement among Japanese workers: a protocol for a cluster randomized controlled trial

**DOI:** 10.1186/s40359-021-00541-6

**Published:** 2021-02-23

**Authors:** Yu Komase, Kazuhiro Watanabe, Norito Kawakami

**Affiliations:** 1 Department of Mental Health, Graduate School of Medicine, The University of Tokyo, 7-3-1 Hongo, Bunkyo-ku, Tokyo, 113-0033 Japan; 2 Japan Society for the Promotion of Science, Chiyoda-ku, Japan

**Keywords:** Gratitude intervention, Occupational health, Positive psychology intervention, Well-being, Work engagement

## Abstract

**Background:**

Work engagement is one of the most important outcomes for both employees and employers. Although the findings to date, integrated 40 intervention studies aiming to improve work engagement, consistent results have not yet been produced, suggesting the importance of further intervention studies. This study aims to investigate the effects of gratitude intervention programs focused on two important work engagement factors among Japanese workers: personal and job resources.

**Methods:**

This study will be a two-arm, parallel-group cluster (organization) randomized control trial. Japanese organizations and nested employees will be recruited through the first author’s acquaintances using snowball sampling. Organizations that meet the inclusion criteria will be randomly allocated to intervention or control groups in a 1:1 ratio within the company unit. The intervention groups will be provided with a 1-month long gratitude intervention program, which aims to promote reciprocal gratitude exchanges within the same organization. The program consists of psychoeducation, gratitude lists, and behavioral gratitude expression. The control groups will not receive any intervention. The primary outcome will be work engagement measured by the Japanese version of the Utrecht Work Engagement Scale at baseline and after 1 (immediate post-survey), 3, and 6 months. Multilevel latent growth modeling will be conducted to examine the effectiveness of the intervention program.

**Discussion:**

This study will be the first cluster randomized controlled trial applied to the investigation of gratitude intervention aimed at improving work engagement among Japanese workers; to promote reciprocal gratitude exchanges within a given organization; and to include both gratitude lists and behavioral gratitude expression. Gratitude interventions have several strengths in terms of implementation: the objectives of the exercises are easy to understand and implement; it does not require much time or expense; they tend to have lower dropout rates; and they do not require experts in psychology. Although implementation difficulties have been common in previous interventions targeting work engagement, gratitude intervention may be suitable even for workers who have limited time to devote to the tasks.

*Trial registration*: This study was registered at the University Hospital Medical Information Network Clinical Trials Registry (UMIN-CTR, ID=UMIN000042546): https://upload.umin.ac.jp/cgi-open-bin/ctr/ctr_view.cgi?recptno=R000048566 on November 25, 2020.

## Background

Work engagement is a positive, fulfilling, work-related state of mind characterized by vigor, dedication, and absorption [1] and is one of the most crucial outcomes for both employees and employers. For employees, high work engagement is positively associated with well-being [2] and physical and mental health [3]. For employers, increased work engagement is linked to high employee performance [4] and low turnover [5]. Therefore, increasing work engagement levels is critical.

Many studies have focused on developing interventions to increase work engagement, and scientific evidence of their effectiveness has been accumulated. According to a systematic review of 40 intervention studies, mindfulness interventions (n = 9) and job crafting (n = 5) were commonly used and proved successful [6]. However, some interventions (n = 6) were ineffective. Because interventions that are consistently effective have not yet been determined, it is important to study other types of interventions [6]. This review also identified job and personal resources as important determinants of work engagement. The Job Demands-Resource model (JD-R model) explains the link between these variables. This model proposes that job and personal resources activate a motivational pathway leading to work engagement and better well-being [7]. Job resources refer to a job's physical, social, or organizational aspects (e.g., social support from supervisor and coworkers). Job resources can reduce job demands (e.g., workload and emotional and cognitive demands), help employees achieve work goals, and stimulate personal learning and development. In comparison, personal resources refer to “positive self-evaluations that are linked to resiliency and refer to individuals’ sense of their ability to control and impact their environment successfully (e.g., self-efficacy and sense of coherence)” [8, 9]. Job and personal resources are important targets for developing work engagement interventions with greater effects.

Gratitude intervention, known as a positive psychology intervention [10], is a potential strategy for increasing these two factors and improving work engagement. Gratitude at work is defined as “the tendency to notice and be thankful for how various aspects of a job affect one's life”; this can be enhanced by regularly recording things for which one is grateful or expressing gratitude to others. Workers with a higher level of gratitude at their job show greater work engagement (0.48 ≤ r ≤ 0.67) [11]. A previous intervention study proposed the “resource building pathway” of gratitude intervention, which encourages the users to build personal resources [12]. This pathway was proposed based on the positive-activity model, which argues that positive activities increase positive emotions, thoughts, and behaviors [13], and combined with “positive self-evaluations,” they increase personal resources. Previous gratitude intervention studies have found significant improvements in personal resources (self-efficacy and optimism) [10, 14, 15]. Although there have been limited intervention studies, a worker's gratitude can be associated with job resources [16, 17]. The pathway can be explained by the broaden-and-build theory [18], which suggests that positive affective states broaden people’s momentary thought–action repertoires. This may help individuals to develop social bonds and enhance perceived social support [19] or engage in help-seeking behaviors.

Moreover, those with increased feelings of gratitude may also promote job resource availability in their workplace through organizational citizenship or prosocial behavior [20–22]. Thus, a gratitude intervention could also improve work engagement. We previously investigated whether individual-level gratitude intervention improved work engagement with a pre-and post-test study design [14] but failed to find a positive effect of intervention. To date, this has been the only study to address the effect of gratitude intervention on work engagement. Further research is needed to understand the effectiveness of gratitude interventions on work engagement using an enhanced program and a better study design, such as a randomized controlled trial (RCT).

Therefore, we developed a new program by modifying two aspects of our original one [14]. First, because the previous program focused on personal resources but ignored job resources, we developed a program that could enhance both resources. Second, we aimed to promote reciprocal gratitude exchanges by increasing the number of workers with higher levels of gratitude within the same organization. Emmons et al. (2016) argued that most of our time is spent at work; because gratitude is a fundamental human requirement, giving and receiving gratitude at work is vital [23]. This approach would expand the program to improve work engagement through personal and job resources that are not viable at the individual level. Receiving gratitude would be useful for enhancing personal resources. A diary study found that receiving gratitude was associated with increased work engagement the following day via self-efficacy improvement [24]. The author argued that receiving gratitude is a positive signal indicating a successful interaction [24]. As noted above, for job resources, workers with higher levels of gratitude tend to provide more support to others and contribute to fostering relational growth and a supportive work environment, increasing job resources in the entire organization. In summary, activating interactions between workers with a higher level of gratitude within the same organization would be useful.

### Study aims and hypothesis

This study aims to develop a modified gratitude intervention program and examine its effect on work engagement among Japanese workers using a cluster randomized controlled (cRCT) design. We hypothesize that gratitude intervention will improve the primary outcome of work engagement in workers in the intervention group. We also hypothesize that intervention will improve gratitude levels at work, personal resources (self-efficacy and sense of coherence), job resources (support from supervisors and coworkers), well-being (eudaimonic well-being and psychological distress), and work performance as secondary outcomes in workers in the intervention group.

## Methods/design

### Study design and setting

This study will be a two-arm, parallel-group, non-blinded cRCT. After completing a baseline survey, randomization will be conducted at the organizational level in a 1:1 ratio for intervention and control groups. An organization refers to a structured social unit of people who are managed to meet specific needs or pursue collective goals. In this study, an organization is a group of departments, sections, or teams working together in a company. Measurements will be collected, and the intervention program’s efficacy will be analyzed, at the individual level, accounting for cluster (organization) level effects. The protocol and this manuscript were written following SPIRIT guidelines [25] and registered at the University Hospital Medical Information Network (UMIN) Clinical Trials Registry (UMIN-CTR, ID=UMIN000042546).

### Participants

This cRCT will include 2 levels: organizations and employees. The intervention program will promote interactions between employees in the same organization by expressing gratitude at each level. There will be no inclusion and exclusion criteria for organizations: any organization wishing to participate in the intervention program may do so. The inclusion criteria for individuals will be workers (1) who are more than twenty years old; (2) who belong to the organizations; (3) who work at the same organization 3 or more days a week; and (4) who have Internet access via smartphones, tablets, or computers. Temporary workers at the target organization who are also employed elsewhere will be included. Exclusion criteria for individuals will not be specified.

### Procedure

**Fig. 1:**
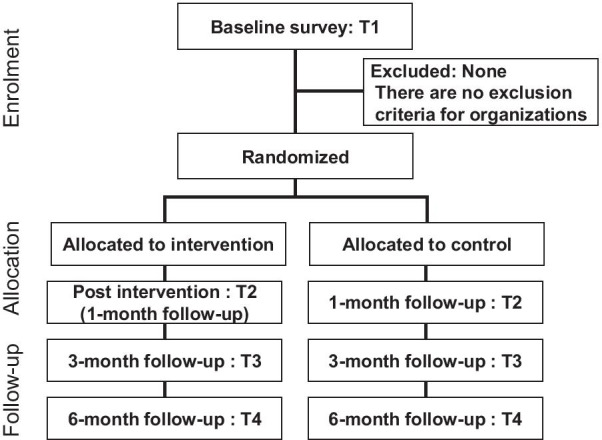
Flow chart of participants

Figure 1 shows a flowchart for participants. Recruitment will be in Japan through acquaintances of the first author (YK) using snowball sampling. Many of the subjects belong to the broad community involved in the University of Tokyo’s occupational mental health field, which comprises more than 300 occupational health professionals, including doctors, nurses, clinical psychologists, and human resources management specialists from different organizations. The first author will invite them to participate in the study. If they agree to assist, the author will ask them to be a coordinator for their company and select organizations for study participation. Then, all nested employees will be recruited for the study. The employees will receive emails from the coordinator providing a program overview and ensuring its voluntary nature. The average cluster size will be approximately 20 employees. After the nested employees complete the baseline survey, organizations will be allocated randomly to the intervention and control groups. The intervention program will last 1 month. Post-surveys will be administered to both intervention and control groups immediately after intervention completion (1-month follow-up) with additional follow-ups at 3 and 6 months.

### Intervention program

**Fig. 2:**
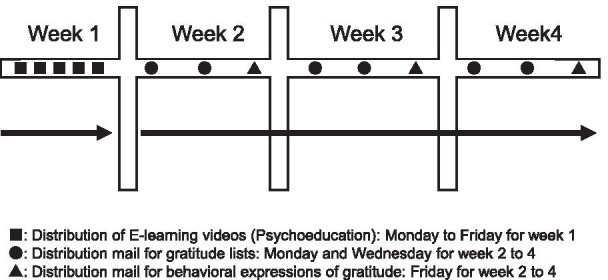
Flow chart of gratitude intervention program

We developed a new gratitude intervention program using the following steps. First, we systematically reviewed RCT studies examining gratitude intervention in workers. The first author then developed a preliminary program in consultation with experts about the occupational stress model, positive psychology, and work engagement. Next, the first author conducted hearing with 5 researchers familiar with gratitude intervention and modified the program according to their recommendations. Lastly, we conducted a 2-week pilot trial with workers nested in one organization (n = 28). The program was finalized after modifications based on the findings. The newly developed gratitude intervention is a 4-week program implemented at an organizational level (Fig. 2). It consists of 3 elements: psychoeducation, gratitude lists, and behavioral gratitude expression (Table 1). Psychoeducation is delivered during week 1 and lasts 1 week. Gratitude lists and behavioral gratitude expressions are completed over the next 3 weeks (weeks 2–4). Gratitude lists and behavioral gratitude expression correspond to increasing personal and job resources, respectively. Psychoeducation will provide a program overview and help participants complete the activities. Details of each component follow.

**Table 1:** Contents of the gratitude intervention program

Elements	Frequency, duration, and timing	Main objectives	Implementation method
Psychoeducation	Frequency: Five times per week (Monday to Friday) Duration: One week Timing: Week 1	1. To understand the expected effects of the program 2. To understand the program's overview and objectives 3. To understand the mechanism of feeling gratitude 4. To understand how to do gratitude lists in this program 5. To understand how to do behavioral expressions of gratitude in this program	Participants will be sent an e-mail with the attached e-learning movie (within 6 min) and a leaflet summarizing the content on a single sheet. Then, they will be asked to watch all movies by the next week
Gratitude lists	Frequency: Twice per week (Monday and Wednesday) Duration: Three weeks Timing: Week 2 to week 4	To enhance personal resources	Participants will be asked to log in to a specific website containing gratitude lists to record what they feel grateful for when thinking about: 1. a supportive work environment 2. meaningful work
Behavioral expressions of gratitude	Frequency: At least once a week (Friday) Duration: Three weeks Timing: Week 2 to week 4	To enhance job resources	Participants will be asked to send a grateful message using instant communication technology, such as e-mail and Facebook, to express their grateful feelings to members in the same organization

#### Psychoeducation

A psychoeducational component has been adopted in several gratitude intervention studies [26, 27]. For example, Froh et al. (2014) provided elementary school students with 5 structured sessions, including understanding a benefactor’s intention when helping a beneficiary and the benefactor’s cost [26]. The current program includes 5 psychoeducational sessions delivered via short videos (≤ 6 min), which will be sent via email. A single-sheet leaflet summarizing the movie’s content will also be attached. The psychoeducational component aims to enhance participants’ motivation, efficacy, beliefs, skills, and interest. These are important factors in positive psychology interventions [28]. If emails with attachments cannot be delivered due to security policies, we will ask the company's coordinator to deliver the video files to the participants. The file contents are summarized in Table 1.

#### Gratitude lists

A gratitude list is a classic strategy requiring participants to list things for which they are regularly grateful [29]. Among workers, gratitude lists significantly improved well-being (life and job satisfaction, positive affect, depressive symptoms, and perceived stress) [30–33] and perceived personal resources (self-efficacy) [14]. In this program, participants will be asked to log in to a website to access gratitude lists and record 2 things for which they are grateful. Two essential components of gratitude at work include gratitude for a supportive work environment and meaningful work [11]. Therefore, participants will be required to record both components each time. Participants will be assured that whatever they record will not be revealed to other organization members. For 3 weeks, they will receive a reminder email twice a week with the website link and asked to log in and record gratitude. In our pilot study, most gratitude entries were completed within 5 min.

#### Behavioral gratitude expression

Behavioral gratitude expression is an intervention that expresses gratitude to someone specific. In the most cited study, participants were asked to write a gratitude letter. They read it face-to-face with their benefactor; this resulted in significant improvement in their well-being [34]. Handwritten letters do not seem to differ from other forms of expression, such as digital forms, in their effectiveness [35]. Some studies have incorporated instant communication technology, such as email or Facebook, rather than letters, to express gratitude [36, 37]. In many studies, participants expressed their gratitude once a week and showed significant improvements in well-being [34, 37, 38]. In this program, participants will be asked to send a message of gratitude using instant communication technology to members of the same organization more than once a week for 3 weeks. They will also be asked to reply to any messages they receive during the program period. These positive workplace interactions should strengthen relationships between employees [22, 39], resulting in increased job resources (support from supervisors or coworkers). In our pilot study, it took 5 min or less to complete 1 message of gratitude.

### Intervention group

The first author will create mailing lists of workers participating in the study for each organization. All program instructions, including psychoeducation, gratitude lists, and behavioral gratitude expression, will be sent through these mailing lists. The program will last 4 weeks; psychoeducation will occur during week 1 and gratitude lists and gratitude expression in weeks 2–4. The first intervention is scheduled to begin January 11, 2021.

### Control group

Participants belonging to the control group will not receive any intervention programs until they complete the 6-month follow-up survey. Then control group participants will receive the intervention program.

### Outcomes

All outcomes will be measured using a self-reported web-based survey at baseline and at 1-month (immediate post-intervention), 3-month, and 6-month follow-ups (Table 2). All outcomes will be calculated at the individual level. The surveys will be administered simultaneously to intervention and control groups at each company.

**Table 2:** Outcome measures

Measurement	Aim	Baseline (T1)	1-month follow-up (T2)	3-month follow-up (T3)	6-month follow-up (T4)
Primary outcome					
UWES	Work engagement	X	X	X	X
Secondary outcome					
GAWS	Gratitude at work	X	X	X	X
GSES	Sense of coherence	X	X	X	X
SOCS	Self-efficacy	X	X	X	X
BJSQ	Coworker support	X	X	X	X
	Supervisor support	X	X	X	X
TOMH well-being-24	Eudaimonic well-being at work	X	X	X	X
K6	Psychological distress	X	X	X	X
WHO-HPQ	Work performance	X	X	X	X

UWES, Utrecht Work Engagement Scale; GAWS, Gratitude At Work Scale; GSES, General Self-Efficacy Scale; SOCS, Sense Of Coherence Scale; BJSQ, Brief Job Stress Questionnaire; TOMH well-being-24, The University of Tokyo Occupational Mental Health well-being scale; K6, Kessler's psychological distress scale; WHO-HPQ; Health Performance Questionnaire

#### Work engagement

As the primary outcome, work engagement will be assessed using the 9-item Japanese version of the Utrecht Work Engagement Scale (UWES) [40]. The UWES has 3 subscales: vigor (3 items, e.g., "At my job, I feel strong and vigorous"), dedication (3 items, e.g., "I am enthusiastic about my job"), and absorption (3 items, e.g., "I am immersed in my work"). All items are measured on a 7-point Likert scale ranging from 0 (Never) to 6 (Always). The reliability and validity of the Japanese UWES were verified previously [40]. The scores for each of the 9 items will be averaged and used for analyses.

#### Gratitude at work

Gratitude at work will be assessed using the Japanese version of the gratitude-at-work scale (GAWS). The scale consists of 2 subscales: gratitude for (1) a supportive work environment and (2) meaningful work. All items are rated on a 5-point Likert-type scale ranging from 1 (Never) to 5 (Always). The reliability and validity of the Japanese GAWS were verified previously [21]. The overall GAWS score will be the average of the 10 items and then used for analyses.

#### Self-efficacy

Self-efficacy will be assessed by the Japanese version of the self-efficacy scale [41]. The scale assesses will to take action, ability to strive for action completion, and patience in adversity. Twenty-three items will be assessed on a 5-point Likert scale ranging from 1 (Disagree) to 5 (Agree). The scale's reliability and validity were confirmed in a previous study [41]. The score will be the average of the 23 items and then used for analyses.

#### Sense of coherence

A sense of coherence (SOC) will be assessed using the Japanese version of the SOC scale [42]. SOC is defined as “individuals’ perceptions of life and resources to help them overcome hardships in life” [42] which relates to stress management ability. The scale consists of three dimensions: comprehensibility, manageability, and meaningfulness. All items are rated on a 7-point Likert-type scale ranging from 1 (Never) to 7 (Always). The Japanese version of the SOC scale's reliability and validity was verified in a previous study [42]. The score will be the average of the 13 items and then used for analyses.

#### Supervisor and coworker support

Supervisor and coworker support will be assessed using the Brief Job Stress Questionnaire (BJSQ) for each of the three items [43]. Items are scored on a 4-point Likert scale from 1 (Not at all) to 4 (Extremely). Higher scores mean higher social support. The scores for each of the 3 items will be averaged and used for analyses.

#### Eudaimonic well-being at work

Eudaimonic well-being at work will be assessed using the 24-item University of Tokyo Occupational Mental Health well-being scale (TOMH well-being 24) [44]. This scale contains 8 factors, such as role-oriented future prospects, autonomy, and role-oriented positive perception, each measured by 3 items. These items are scored on a 7-point scale ranging from 0 (Strongly disagree) to 6 (Strongly agree). The scale's reliability and validity were confirmed previously [44]. The scores from each of the 24 items were averaged and used for analyses.

#### Psychological distress

Psychological distress will be measured with the Japanese version of K6, which asks respondents how frequently they had experienced symptoms of psychological distress during the previous 30 days using 6 items [45]. Responses are rated on a 5-point Likert scale ranging from 0 (None of the time) to 4 (All of the time). A total score of these items (0 to 24) will be calculated and used for analyses.

#### Work performance

Work performance will be assessed using the validated Japanese short version of the WHO Health and Work Performance Questionnaire (WHO-HPQ) [46]. The scale includes one item that scores an individual’s overall job performance over the past month on a scale from 0 (Worst) to 10 (Best). The ratings will be multiplied by 10 to calculate work performance according to WHO-HPQ scoring guidelines.

### Sample size calculation

The target sample size was calculated by accounting for intraclass correlations (ICC) of the organizations’ outcomes, according to the guidelines in the Consolidated Standards of Reporting Trials (CONSORT) for cRCTs [47]. Sample sizes in cRCTs should be multiplied by design effect (1 + [m − 1]ρ), where m is the average cluster size, and ρ is ICC. In a previous study, ICC for work engagement among Japanese employees was 0.09 [48]. The cluster size was set to 20 [49]. An effect size of the individual intervention program was estimated at 0.30 based on a previous meta-analysis on well-being [39, 50]. Based on these estimations, 637 participants from 32 organizations per arm will be needed at an alpha error rate of 0.05 and a beta error rate of 0.10 using G*Power version 3.1.9.2 [51, 52].

### Randomization

Companies participating in the study will select organizations to participate in the program. Although the coordinator will choose the organizations, if necessary, the first author and the coordinator will discuss how to select or classify the organization. The selected organizations will then be randomly assigned to the intervention or control group in a 1:1 ratio within the company unit. Permuted block randomization (block size = 2) will be adopted for equal randomization. For example, if 4 organizations in a company participate, 2 will be assigned to the intervention group and 2 to the control group. Assignments will be made after all participating employees have responded to the baseline survey (T1). All data related to randomization (company and organization lists and random number tables) will be password-protected. Research assistants not involved in program administration or results analyses and who are blind to the researchers (YK, KW, and NK) will carry out the entire randomization process. Because of the nature of the intervention, it is not possible to blind the person implementing it.

#### Statistical analysis

Primary analysis of the intervention program’s effects on work engagement will be carried out by multilevel latent growth modeling (LGM) [53] using robust maximum likelihood estimation. This study will examine repeated measures for employees at level 1, work engagement within employees at level 2, and interactions within organizations at level 3. We will compare the significance of the dummy-variable coefficient for the intervention (control = 0, intervention = 1), with the linear slope of work engagement representing the intervention program’s effect. We will reference some model fit indices, such as χ^2^, the comparative fit index (CFI), the Tucker–Lewis index (TLI), and root mean square error of approximation (RMSEA). We will then determine the model that demonstrates a good fit based on the fit indices, specifically CFI and TLI greater than 0.95 and RMSEA smaller than 0.06 [54]. Intention-to-treat (ITT) analysis using full information maximum likelihood estimation will be conducted, including all employees who complete the baseline survey. If the LGM yields misspecified or improper solutions, we will consider running a three-level mixed-model analysis using restricted maximum likelihood estimation. If no data hierarchy is found in the organizational analysis (e.g., ICC is not significant or less than 0.05) [55], we will consider analyzing the data individually. Mplus Version 8 [56] and PASW Statistics 20 will be used for LGM and mixed model analysis, respectively. The analysis of secondary outcomes (gratitude at work, self-efficacy, sense of coherence, support from supervisors and coworkers, eudaimonic well-being at work, psychological distress, and work performance) will be conducted using the same methods. Potential subgroup analyses will be conducted, stratified by degree of commitment to the program (numbers of gratitude lists and behavioral gratitude expressions) and initial work engagement levels or gratitude at work. Some mediation analyses will also be conducted using multilevel structural equation modeling.

#### Data monitoring

Due to limited human resources, a Data Monitoring Committee (DMC) consisting of the first author (YK) and coordinator will be established at each organization. The DMC will discuss at each organization three months after randomization to review the participation rates and reasons for study dropouts. Data and Safety Monitoring Board (DSMB) will be prepared to monitor recruitment progress and data collection (e.g., percentage completing each follow-up) independently of sponsors and without competing interests.

#### Patient and public involvement statement

Partnering with people involved in the study is an ethical imperative for improving the study’s quality. In this study, working employees will be the intervention’s main target, and their collaboration in the process of program development will be important. In this context, we conducted a preliminary study to seek input from workers, which was reflected in the intervention program. However, these workers were not involved in the conception, design, and writing of the protocol paper.

#### Dissemination of research findings

This study’s findings will be submitted to peer-reviewed journals for publication following CONSORT guidelines for cRCTs. Participants will be notified of conference presentations and publications.

## Discussion

To the best of our knowledge, this study will be the first cRCT to investigate the effects of gratitude intervention on work engagement. Compared with other psychological interventions, gratitude interventions have several strengths in terms of implementation: the objectives of the exercises are easy to understand and implement; it does not require much time or expense; they tend to have lower dropout rates; and they do not require experts in psychology [39, 57]. Although implementation difficulties have been common in previous interventions targeting work engagement [6], gratitude intervention may be suitable even for workers who have limited time to devote to the tasks.

This study will have several limitations. First, because all data will be collected using a self-reported questionnaire, measurement errors and information bias could be introduced. Second, generalizability will be limited because snowball sampling will be used instead of random sampling. Third, due to the intervention's nature, it is not possible for both the intervention implementer and participants to be blind to group assignments.

## Data Availability

Upon publication of the results, the datasets generated and/or analysed during the current study will be available from the corresponding author on reasonable request.

## Abbreviations

JD-R model: Job demands-resource model
RCT: Randomized controlled trial
UMIN-CTR: University Hospital Medical Information Network Clinical Trials Registry
UWES: Utrecht Work Engagement Scale
GAWS: Gratitude At Work Scale
GSES: General Self-Efficacy Scale
SOCS: Sense Of Coherence Scale
BJSQ: Brief Job Stress Questionnaire
TOMH well-being-24: The University of Tokyo Occupational Mental Health well-being scale
K6: Kessler's psychological distress scale
WHO-HPQ: Health Performance Questionnaire
ICC: Intraclass correlations
CONSORT: Consolidated Standards of Reporting Trials
LGM: Latent growth modeling
CFI: Comparative fit index
TLI: Tucker–Lewis index
RMSEA: Root mean square error of approximation
ITT: Intention-to-treat
DMC: Data Monitoring Committee
DSMB: Data and Safety Monitoring Board

## References

[1] Schaufeli WB, Salanova M, González-Romá V, Bakker AB (2002). The measurement of engagement and burnout: a two sample confirmatory factor analytic approach. J Happiness Stud.

[2] Schaufeli WB, Taris TW, Rhenen WV (2008). Workaholism, burnout, and work engagement: three of a kind or three different kinds of employee well-being?. Appl Psychol.

[3] Leijten FRM, Heuvel SG, Beek AJ (2015). Associations of work-related factors and work engagement with mental and physical health: a 1-year follow-up study among older workers. J Occup Rehabil.

[4] Christian MS, Garza AS, Slaughter JE (2011). Work engagement: a quantitative review and test of its relations with task and contextual performance. Pers Psychol.

[5] Plooy JD, Roodt G (2010). Work engagement, burnout and related constructs as predictors of turnover intentions. SA J Ind Psychol.

[6] Knight C, Patterson M, Dawson J (2019). Work engagement interventions can be effective: a systematic review. Eur J Work Organ Psychol.

[7] Bakker AB, Demerouti E (2007). The job demands-resources model: state of the art. J Manag Psychol.

[8] Bakker AB, Demerouti E (2008). Towards a model of work engagement. Career Dev Int.

[9] Vogt K, Hakanen JJ, Jenny GJ, Bauer GF (2016). Sense of coherence and the motivational process of the job-demands-resources model. J Occup Health Psychol.

[10] Emmons RA, McCullough ME (2003). Counting blessings versus burdens: an experimental investigation of gratitude and subjective well-being in daily life. J Pers Soc Psychol.

[11] Cain IH, Cairo A, Duffy M (2018). Measuring gratitude at work. J Posit Psychol.

[12] Heckendorfa H, Lehra D, Ebertb DD, Freund H (2019). Efficacy of an internet and app-based gratitude intervention in reducing repetitive negative thinking and mechanisms of change in the intervention's effect on anxiety and depression: results from a randomized controlled trial. Behav Res Ther.

[13] Lyubomirsky S, Layous K (2013). How do simple positive activities increase wellbeing?. Curr Dir Psychol Sci.

[14] Komase Y, Watanabe K, Imamura K, Kawakami N (2019). Effects of a newly developed gratitude intervention program on work engagement among Japanese workers. J Occup Environ Med.

[15] Klibert J, Rochani H, Samawi H, LaBarge KL, Ryan R (2019). The impact of an integrated gratitude intervention on positive affect and coping resources. Int J Appl Posit Psychol.

[16] Hu X, Kaplan S (2014). Is ‘‘feeling good’’ good enough? differentiating discrete positive emotions at work. J Organ Behav.

[17] Fabio AB, Palazzeschi L, Bucci O (2017). Gratitude in organizations: a contribution for healthy organizational contexts. Front Psychol.

[18] Fredrickson BL (2001). The role of positive emotions in positive psychology. The broaden-and-build theory of positive emotions. Am Psychol.

[19] Lin CC (2016). The roles of social support and coping style in the relationship between gratitude and well-being. Pers Ind Dif.

[20] Spence JR, Brown DJ, Keeping LM, Lian H (2013). Helpful today, but not tomorrow? Feeling grateful as a predictor of daily organizational citizenship behaviors. Pers Psychol.

[21] Komase Y, Watanabe K, Sasaki N, Kawakami N (2020). Reliability and validity of the Japanese version of the gratitude at work scale (GAWS). J Occup Health.

[22] Ma LK, Tunney RJ, Ferguson E (2017). Does gratitude enhance prosociality?: A meta-analytic review. Psychol Bull.

[23] Emmons R. The Little Book of Gratitude: Gaia, 2016.

[24] Lee HW, Bradburn J, Johnson RE, Lin SH, Chang CH (2019). The benefits of receiving gratitude for helpers: a daily investigation of proactive and reactive helping at work. J Appl Psychol.

[25] Chan AW, Tetzlaff JM, Altman DG (2013). SPIRIT 2013 statement: defining standard protocol items for clinical trials. Ann Intern Med.

[26] Froh JJ, Bono G, Emmons RA (2014). Nice thinking! an educational intervention that teaches children how to think gratefully. School Psych Rev.

[27] Owens RL, Patterson MM (2013). Positive psychological interventions for children: a comparison of gratitude and best possible selves approaches. J Genet Psychol.

[28] Sheldon KM, Lyubomirsky S (2007). Is it possible to become happier? (and if so, how?). Soc Personal Psychol Compass.

[29] Wood AM, Froh JJ, Geraghty AW (2010). Gratitude and well-being: a review and theoretical integration. Clin Psychol Rev.

[30] Chan DW (2013). Counting blessings versus misfortunes: positive interventions and subjective well-being of Chinese school teachers in Hong Kong. Educ psychol.

[31] Kaplan S, Bradley GJC, Ahmad A (2014). A test of two positive psychology interventions to increase employee well-being. J Bus Psychol.

[32] Cheng ST, Ki TP, Lam JH (2015). Improving mental health in health care practitioners: randomized controlled trial of a gratitude intervention. J Consult Clin Psychol.

[33] Neumeier LM, Brook L, Ditchburn G, Sckopke P (2017). Delivering your daily dose of well-being to the workplace: a randomized controlled trial of an online well-being programme for employees. Eur J Work Organ Psychol.

[34] Seligman MEP, Steen TA, Park N, Peterson C (2005). Positive psychology progress: empirical validation of interventions. Am Psychol.

[35] Harlyey J, Sotto R, Pennebaker J (2003). Speaking versus typing: a case study of the effects of using voice-recognition software on academic correspondence. Br J Educ Technol.

[36] Renshaw TL, Hindman ML (2017). Expressing gratitude via instant communication technology: a randomized controlled trial targeting college students’ mental health. Ment Health Prev.

[37] O’Connell BH, O’Shea D, Gallagher S (2017). Feeling thanks and saying thanks: a randomized controlled trial examining if and how socially oriented gratitude journals work. J Clin Psychol.

[38] Toepfer SM, Cichy K, Peters P (2012). Letters of Gratitude: Further Evidence for Author Benefits. J Happiness Stud.

[39] Davis D, Choe E, Meyers J (2016). Thankful for the little things: a meta-analysis of gratitude interventions. J Couns Psychol.

[40] Shimazu A, Schaufeli WB, Kosugi S (2008). Work engagement in Japan: validation of the Japanese version of the Utrecht Work Engagement Scale. Appl Psychol.

[41] Narita K, Shimonaka Y, Nakazato K (1995). A Japanese version of the generalized self-efficacy scale: scale utility from the life span perspective. Japanese J Educ Psychol.

[42] Antonovsky A. Unraveling the Mystery of Health: How People Manage Stress and Stay Well [Kenko-no-nazo-wo-toku] Trans Yamazaki Y, Yoshii K. Tokyo: Yushindo Kobunsha; 1987.

[43] Shimomitsu T, Haratani T, Nakamura K, Kato M (2000). Final development of the Brief Job Stress Questionnaire mainly used for assessment of the individuals. The Ministry of Labor Sponsored Grant for the prevention of work-related illness.

[44] Watanabe K, Imamura K, Inoue A (2020). Measuring eudemonic well-being at work: a validation study for the 24-item The University of Tokyo Occupational Mental Health well-being scale among Japanese workers. Ind Health.

[45] Furukawa TA, Kawakami N, Saitoh M (2008). The performance of the Japanese version of the K6 and K10 in the World Mental Health Survey Japan. Int J Methods Psychiatr Res.

[46] Kessler RC, Barber C, Beck A (2003). The World Health Organization Health and Work Performance Questionnaire (HPQ). J Occup Environ Med.

[47] Campbell MK, Elbourne DR, Altman DG (2004). CONSORT statement: extension to cluster randomised trials. BMJ.

[48] Kunie K, Kawakami N, Shimazu A, Yonekura Y, Miyamoto Y (2017). The relationship between work engagement and psychological distress of hospital nurses and the perceived communication behaviors of their nurse managers: a cross-sectional survey. Int J Nurs Stud.

[49] LaMontagne AD, Milner AJ, Allisey AF (2016). An integrated workplace mental health intervention in a policing context: protocol for a cluster randomised control trial. BMC Psychiatry.

[50] Dickens LR (2017). Using gratitude to promote positive change: a series of meta-analyses investigating the effectiveness of gratitude interventions. Basic Appl Soc Psych.

[51] Faul F, Erdfelder E, Lang AG (2007). G*Power 3: a flexible statistical power analysis program for the social, behavioral, and biomedical sciences. Behav Res Methods.

[52] Faul F, Erdfelder E, Buchner A (2009). Statistical power analyses using G*Power 3.1: tests for correlation and regression analyses. Behav Res Methods..

[53] Jackson DL (2010). Reporting results of latent growth modeling and multilevel modeling analyses: some recommendations for rehabilitation psychology. Rehab Psychol.

[54] Hu L, Bentler PM (1999). Cutoff criteria for fit indexes in covariance structure analysis: conventional criteria versus new alternatives. Struct Equ Modeling.

[55] Raudenbush SW, Anthony SB (2001). Hierarchical linear models: applications and data analysis.

[56] Muthén LK, Muthén BO. Mplus user’s guide. 7th edition. Los Angeles, CA, 1998–2017. https://www.statmodel.com/ugexcerpts.Shtml

[57] Geraghty AWA, Wood AM, Hyland ME (2010). Attrition from self-directed interventions: investigating the relationship between psychological predictors, intervention content and dropout from a body dissatisfaction intervention. Soc Sci Med.

